# Development and Validation of an Esophageal Squamous Cell Carcinoma Detection Model by Large-Scale MicroRNA Profiling

**DOI:** 10.1001/jamanetworkopen.2019.4573

**Published:** 2019-05-24

**Authors:** Kazuki Sudo, Ken Kato, Juntaro Matsuzaki, Narikazu Boku, Seiichiro Abe, Yutaka Saito, Hiroyuki Daiko, Satoko Takizawa, Yoshiaki Aoki, Hiromi Sakamoto, Shumpei Niida, Fumitaka Takeshita, Takahiro Fukuda, Takahiro Ochiya

**Affiliations:** 1 Department of Breast and Medical Oncology, National Cancer Center Hospital, Tokyo, Japan; 2 Advanced Clinical Research of Cancer, Juntendo University Graduate School of Medicine, Tokyo, Japan; 3 Department of Gastrointestinal Medical Oncology, National Cancer Center Hospital, Tokyo, Japan; 4 Division of Molecular and Cellular Medicine, National Cancer Center Research Institute, Tokyo, Japan; 5 Endoscopy Division, National Cancer Center Hospital, Tokyo, Japan; 6 Department of Esophageal Surgery, National Cancer Center Hospital, Tokyo, Japan; 7 Toray Industries, Inc, Kamakura, Japan; 8 Dynacom Co, Mihama, Japan; 9 Department of Biobank and Tissue Resources, National Cancer Center Research Institute, Tokyo, Japan; 10 Medical Genome Center, National Center for Geriatrics and Gerontology, Obu, Japan; 11 Fundamental Innovative Oncology Core Center, National Cancer Center Research Institute, Tokyo, Japan; 12 Department of Hematopoietic Stem Cell Transplantation, National Cancer Center Hospital, Tokyo, Japan

## Abstract

**Importance:**

Patients with late-stage esophageal squamous cell carcinoma (ESCC) have a poor prognosis. Noninvasive screening tests using serum microRNAs (miRNAs) to accurately detect early-stage ESCC are needed to improve mortality.

**Objective:**

To establish a model using serum miRNAs to distinguish patients with ESCC from noncancer controls.

**Design, Setting, and Participants:**

In this case-control study, serum miRNA expression profiles of patients with ESCC (n = 566) and control patients without cancer (n = 4965) were retrospectively analyzed to establish a diagnostic model, which was tested in a training set and confirmed in a validation set. Patients histologically diagnosed as having ESCC who did not receive prior therapy or have a past or concurrent cancer other than ESCC were enrolled from the National Cancer Center Hospital in Tokyo, Japan. Between October 2010 and November 2015, control samples were collected from the National Cancer Center Biobank, the Biobank of the National Center for Geriatrics and Gerontology, and the general population undergoing routine health examination. Data analysis was performed between August 2015 and October 2018. Serum samples were randomly divided into discovery and validation sets.

**Main Outcomes and Measures:**

The expression of 2565 miRNAs was assessed in each sample. The discriminant model (named the EC index) was evaluated in the training set using Fisher linear discriminant analysis with a greedy algorithm. Receiver operating characteristic curve analysis evaluated the diagnostic ability of the model in the validation set.

**Results:**

In the training set, 283 patients with esophageal cancer (median age, 67 years [range, 37-90 years]; 83.4% male) were compared with 283 control patients (median age, 54 years [range, 22-100 years]; 43.1% male), and the EC index was constructed using 6 miRNAs (miR-8073, miR-6820-5p, miR-6794-5p, miR-3196, miR-744-5p, and miR-6799-5p). The area under the receiver operating characteristic curve was 1.00, with sensitivity of 1.00 and specificity of 0.98. The validation set included 283 patients (median age, 66 years [range, 42-87 years]; 83.0% male) and 4682 control patients (median age, 68 years [range, 20-98 years]; 44.7% male), and the area under the receiver operating characteristic curve for the EC index was 1.00, with sensitivity of 0.96 and specificity of 0.98.

**Conclusions and Relevance:**

What appears to be novel serum miRNA discriminant model was developed for the diagnosis of ESCC. A multicenter prospective study is ongoing to confirm the present observations.

## Key Points

**Question:**

Can circulating microRNAs be used as biomarkers to detect esophageal squamous cell carcinoma?

**Findings:**

In this case-control study of 566 patients with esophageal squamous cell carcinoma and 4965 control patients without cancer, serum samples from patients with esophageal squamous cell carcinoma and control patients were used to establish a model to detect esophageal squamous cell carcinoma using 6 microRNAs in a training set. In the validation set, the model distinguished patients with cancer from control patients with high sensitivity and high specificity (0.96 and 0.98, respectively).

**Meaning:**

The 6-microRNA model is a promising noninvasive screening tool in esophageal squamous cell carcinoma.

## Teaser

This case-control study establishes and assesses the validity of a model using serum microRNAs (miRNAs) to distinguish patients with esophageal squamous cell carcinoma (SCC) from patients without cancer.

## Introduction

In 2012, the worldwide incidence of and number of deaths from esophageal cancer were estimated at 455 800 and 400 200, respectively.^1^ There are 2 histological types of esophageal cancer, squamous cell carcinoma and adenocarcinoma. Esophageal squamous cell carcinoma (ESCC) is the most common histological type in most geographical areas, including eastern Asia and eastern and southern Africa, where the incidence of esophageal cancer is high. In Japan, the age-adjusted incidence of esophageal cancer was 31.0 cases per 100 000 men and 5.6 cases per 100 000 women per year in 2013, and 90.5% of them were squamous cell carcinomas.^2,3^

The 5-year overall survival rates after surgery are 80.5% for stage I ESCC, 62.2% for stage II, 38.8% for stage III, 22.8% for stage IVA, and 14.2% for stage IVB.^3^ The prognosis of patients is negatively correlated with stage. Patients with early-stage disease may be cured by endoscopic treatment, and patients without distant metastasis are eligible for curative surgery or definitive chemoradiotherapy. According to the Japanese esophageal cancer registry, 68.1% of patients with stage IA receive curative treatment with endoscopy,^3^ underscoring the importance of early detection to reduce the mortality of ESCC. However, to date there are no population screening tests for ESCC that are recommended. Although esophagogastroduodenoscopy is considered the most sensitive method for diagnosing ESCC, it is not recommended as a population screening test for ESCC because of its invasiveness and cost.

Several studies have investigated the role of circulating microRNAs (miRNAs) as diagnostic biomarkers. The miRNAs are noncoding RNAs composed of 19 to 24 nucleotides, and they serve as a hub in gene regulatory networks that control numerous targets through RNA silencing and posttranscriptional regulation of gene expression.^4^ In addition, miRNAs are involved in many biological activities, including cancer development. Furthermore, circulating miRNAs are stable,^5^ and prolonged storage at room temperature and freeze-thawing have a minimal influence on miRNA expression levels.^6^ Circulating miRNA tests are less invasive than other methods and are thus good candidates for cancer screening tests.

Our group is involved in a project to develop a new test based on the use of circulating miRNAs to diagnose 13 cancers, including ESCC, gastric cancer, colorectal cancer, pancreatic cancer, liver cancer, biliary tract cancer, lung cancer, breast cancer, ovarian cancer, prostate cancer, bladder cancer, bone and soft-tissue tumors, and glioma. The test uses a miRNA panel (3D-Gene Human miRNA Oligo Chip; Toray Industries, Inc) to measure the expression of 2565 miRNAs. In this case-control study, we report the results of a study aimed at developing a model to differentiate between patients with ESCC and control patients without cancer using serum miRNAs.

## Methods

### Study Population

Serum samples from 566 patients with ESCC and without other cancers obtained from the National Cancer Center Hospital (NCCH) in Tokyo, Japan, between 2008 and 2014 were retrospectively analyzed. Serum samples were obtained before patients underwent treatment and stored at −20°C in the National Cancer Center Biobank. Control patients without cancer included the following: (1) 323 patients with benign diseases and no cancer treated at the NCCH between 2008 and 2016 (noncancer 1); (2) 2670 individuals whose serum samples were stored at −80°C in the Biobank of the National Center for Geriatrics and Gerontology (NCGG) in Obu, Japan, between 2012 and 2016 (noncancer 2); and (3) 1972 healthy volunteers older than 35 years whose serum samples were collected in 2015 and stored at −80°C at Minoru Clinic in Yokohama, Japan (noncancer 3).

**Figure 1.**
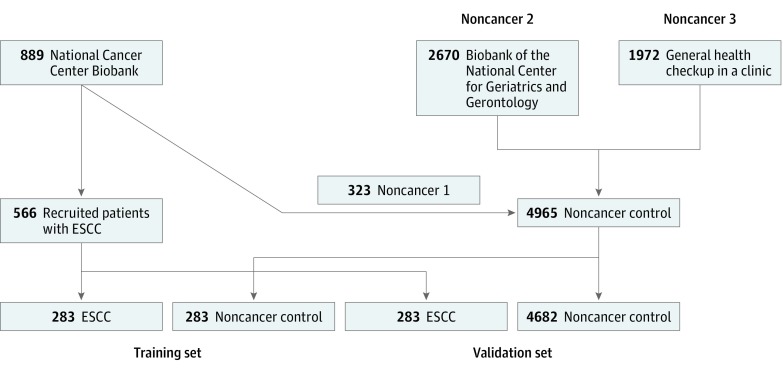
Flowchart of the Development of the EC Index Noncancer control serum samples were collected from the National Cancer Center Biobank (noncancer 1), the Biobank of the National Center for Geriatrics and Gerontology (noncancer 2), and the general population undergoing routine health examinations at a clinic in Yokohama, Japan (noncancer 3). ESCC indicates esophageal squamous cell carcinoma.

The present study involving human participants was approved by the NCCH Institutional Review Board, the Ethics and Conflict of Interest Committee of the NCGG, and the Research Ethics Committee of Medical Corporation Shintokai Yokohama Minoru Clinic. Patients with cancer and control patients without cancer provided written informed consent for the use of serum samples for research purposes. The study followed the Strengthening the Reporting of Observational Studies in Epidemiology (STROBE) reporting guideline. Data analysis was performed between August 2015 and October 2018. Samples from patients with ESCC were randomly divided into a training set and a validation set at a 1:1 ratio (283:283) (Figure 1). The noncancer control group in the training set consisted of 283 participants, including 162 from noncancer control 1, 61 from noncancer control 2, and 60 from noncancer control 3. The noncancer control group in the validation set consisted of 4682 participants, including 161 from noncancer control 1, 2609 from noncancer control 2, and 1912 from noncancer control 3.

### miRNA Extraction and Expression Analysis

Total RNA was extracted from 300 μL of serum using the 3D-Gene RNA extraction reagent from a liquid sample kit (Toray Industries, Inc) and concentrated. Fluorescent labeling of RNA was performed using a 3D-Gene miRNA Labeling kit. RNA was hybridized to the 3D-Gene Human miRNA Oligo Chip designed to detect 2565 miRNA sequences registered in miRBase release 21 (http://www.mirbase.org/). The chip was scanned using a 3D-Gene Scanner (Toray Industries, Inc). The miRNAs with a signal higher than the background signal were selected in advance (positive call), and the background signal was subtracted from each positive call miRNA signal. Only preprocessed positive call miRNAs were used for subsequent analyses. The miRNA signal values were standardized to the ratio of the average signal value of internal control miRNAs (miR-149-3p, miR-2861, and miR-4463) that were stably detected in more than 500 serum samples to normalize the signals across the different microarrays.^7^ To identify robust miRNAs, those with a normalized signal value exceeding 64 in more than 50% of the samples in each group were selected. Microarray data included in the study were obtained in accord with the Minimum Information About a Microarray Experiment guidelines.^8^ Data sets analyzed in the study were submitted to the National Center for Biotechnology Information Gene Expression Omnibus database under accession number GSE122497.

### Statistical Analysis

In the training set, serum miRNA expression profiles were compared between patients with ESCC and control patients without cancer to establish a diagnostic model using Fisher linear discriminant analysis with a greedy algorithm. In the greedy algorithm, the top 20 miRNAs were initially selected based on the discrimination accuracy with leave-1-out cross-validation. Then, each remaining miRNA was added to the selected 20 miRNAs, and the top 20 combinations of 2 miRNAs were selected based on the discrimination accuracy with leave-1-out cross-validation (eMethods in the Supplement). This procedure was repeated to construct discriminant models using up to 8 miRNAs. Finally, the best combination models for each number of miRNAs were compared, and the discriminant model (named the EC index) showing high accuracy with a small number of miRNAs was selected.

Receiver operating characteristic curve analysis was performed to evaluate the diagnostic accuracy of the model in the validation set. Subgroup analysis was performed for each control group to determine the association of differences in sample conditions (ie, tumor location and storage temperature) with specificity. Subgroup analysis was also performed for each cancer stage of patients with ESCC to determine the association of stage with sensitivity. Clinical information of patients with ESCC was collected from the hospital cancer registry. In the in-hospital cancer registry, the *International Union Against Cancer*, 7th edition clinical stage classification was used for patients who had a diagnosis of ESCC in 2012 onward, and the 6th edition was used for patients who had a diagnosis before 2012. The clinicopathologic characteristics of the training set and validation set were compared using the *t* test for continuous variables and Pearson χ^2^ test for categorical variables.

Statistical analyses were performed using the following software programs: R version 3.1.2 (R Project for Statistical Computing), compute.es package version 0.2-4, glmnet package version 2.0-3, hash package version 2.2.6, MASS package version 7.3-45, mutoss package version 0.1-10, pROC package version 1.8, and IBM SPSS Statistics version 23 (IBM Japan). Unsupervised clustering and heat map generation with sorted data sets using Pearson product moment correlation and the method for linkage analysis by Ward^9^ were performed with Partek Genomics Suite 6.6. Principal component analysis was also performed using Partek Genomics Suite 6.6. The limit of statistical significance for all analyses was defined as a 2-sided *P* = .05.

## Results

### Characteristics of Patients With ESCC and Controls

The characteristics of 566 patients with ESCC and 4965 controls in the training and validation sets are listed in Table 1. The training set consisted of 283 patients with ESCC (median age, 67 years [range, 37-90 years]; 83.4% male) and 283 control patients without cancer (median age, 54 years [range, 22-100 years]; 43.1% male), and the validation set consisted of 283 patients with ESCC (median age, 66 years [range, 42-87 years]; 83.0% male) and 4682 control patients (median age, 68 years [range, 20-98 years]; 44.7% male). More than 50% (162 of 283 in the training set and 144 of 283 in the validation set) of patients with ESCC had stage 0 or I disease. Most of the individuals from the NCGG (2586 of 2670) were older than 60 years, and approximately half of them (1469 of 2670) were diagnosed as having dementia. Control patients without cancer from the NCCH had benign breast disease (n = 33) or benign bone or soft-tissue tumors (n = 290).

**Table 1.** Clinicopathologic Characteristics of Study Participants

Covariate	Training Set (n = 283)	Validation Set (n = 283)	*P* Value
**ESCC**			
Total No. (%) of controls	283 (100)	283 (100)	NA
Age, median (range), y	67 (37-90)	66 (42-87)	.17^a^
Sex, No. (%)			
Male	236 (83.4)	235 (83.0)	.91^b^
Female	47 (16.6)	48 (17.0)	
Squamous cell carcinoma, No. (%)	283 (100)	283 (100)	NA
Tumor location, No. (%)			
Cervical esophagus	7 (2.5)	14 (4.9)	.29^b^
Thoracic esophagus	264 (93.3)	258 (91.2)	
Abdominal esophagus	12 (4.2)	11 (3.9)	
Stage, No. (%)			
0	23 (8.1)	28 (9.9)	.30^b^
I	139 (49.1)	116 (41.0)	
II	52 (18.4)	63 (22.3)	
III	59 (20.8)	69 (24.4)	
IV	10 (3.5)	7 (2.5)	
**Control Patients Without Cancer**			
Total No. (%) of controls	283 (100)	4682 (100)	NA
Age, median (range), y	54 (22-100)	68 (20-98)	<.001^a^
Sex, No. (%)			
Male	122 (43.1)	2095 (44.7)	.59^b^
Female	161 (56.9)	2587 (55.3)	
Type of control, No. (%)			
National Cancer Center Biobank benign	162 (57.2)	161 (3.4)	<.001^b^
Biobank of the National Center for Geriatrics and Gerontology	61 (21.6)	2609 (55.7)	
General health checkup in a clinic	60 (21.2)	1912 (40.8)	

Abbreviations: ESCC, esophageal squamous cell carcinoma; NA, not applicable.

^a^ By *t* test.

^b^ By Pearson χ^2^ test.

### Development of the EC Index

**Figure 2.**
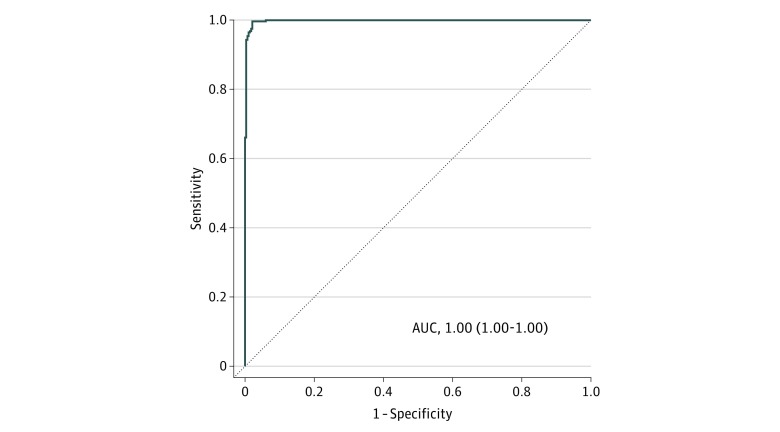
Receiver Operating Characteristic Curve Analysis of a 6-MicroRNA Model (Named the EC Index) in the Training Set AUC indicates area under the curve. Values in parentheses indicate 95% CI.

Among 2565 miRNAs, 343 had a normalized signal value exceeding 64 in more than 50% of samples in each group. eTable 1 in the Supplement lists the top 20 combination models for each number of miRNAs in the training set, and Table 2 lists the best combination models for each number of miRNAs. We selected a model based on 6 miRNAs (miR-8073, miR-6820-5p, miR-6794-5p, miR-3196, miR-744-5p, and miR-6799-5p) as the EC index because the model achieved a high accuracy of 0.99 using the fewest miRNAs. The EC index had a sensitivity and specificity of 1.00 and 0.98, respectively. The EC index was calculated as follows: (0.961037)*miR-8073+(−0.962054)*miR-6794-5p+(1.31647)*miR-3196+(−1.0132)*miR-6820-5p+(0.657628)*miR-744-5p+(−0.406723)*miR-6799-5p-9.799262. eFigure 1 in the Supplement shows the ability of each miRNA in the EC index to distinguish between ESCC and controls in the training set. In the training set, receiver operating characteristic curve analyses showed that each miRNA achieved moderate to low accuracy for discrimination (area under the curve [AUC], 0.64-0.88) (eFigure 2 in the Supplement). The EC index that included 6 miRNAs showed significantly better diagnostic accuracy than each miRNA alone (AUC, 1.00; 95% CI, 1.00-1.00) (Figure 2).

**Table 2.** Best Combination Models of MicroRNAs in the Training Set

No. of MicroRNAs	Model Candidates	Training Set			
		Sensitivity (95% CI)	Specificity (95% CI)	Accuracy (95% CI)	AUC (95% CI)
1	(−1.81483)*miR-6800-5p+14.33326	0.90 (0.86-0.93)	0.76 (0.71-0.81)	0.83 (0.80-0.86)	0.90 (0.87-0.92)
2	(1.02272)*miR-8073+(−2.17698)*miR-6794-5p+10.0051	0.90 (0.87-0.94)	0.94 (0.92-0.97)	0.92 (0.90-0.94)	0.98 (0.97-0.99)
3	(1.09219)*miR-8073+(−2.28576)*miR-6794-5p+(0.826 147)*miR-3180-3p+3.06847	0.95 (0.92-0.97)	0.95 (0.93-0.98)	0.95 (0.93-0.97)	0.98 (0.97-0.99)
4	(0.996309)*miR-8073+(−1.47812)*miR-6794- 5p+(1.20479)*miR-4665-5p+(−1.08956)*miR-6820- 5p+1.83975	0.96 (0.94-0.98)	0.97 (0.95-0.99)	0.96 (0.95-0.98)	0.99 (0.99-1.00)
5	(0.9634)*miR-8073+(−1.07335)*miR-6794- 5p+(1.36201)*miR-3196+(−1.06632)*miR-6820- 5p+(0.576916)*miR-744-5p-11.99418	0.99 (0.98-1.00)	0.98 (0.96-0.99)	0.98 (0.97-0.99)	1.00 (0.99-1.00)
6	(0.961037)*miR-8073+(−0.962054)*miR-6794- 5p+(1.31647)*miR-3196+(−1.0132)*miR-6820- 5p+(0.657628)*miR-744-5p+(−0.406723)*miR-6799-5p- 9.799262	1.00 (0.99-1.00)	0.98 (0.96-1.00)	0.99 (0.98-1.00)	1.00 (1.00-1.00)
7	(1.04095)*miR-8073+(−1.08968)*miR-6794- 5p+(1.63487)*miR-3196+(−0.973975)*miR-6820- 5p+(0.542712)*miR-744-5p+(0.607937)*miR-4433a- 3p+(−0.267989)*miR-4734-17.29468	0.99 (0.98-1.00)	0.99 (0.98-1.00)	0.99 (0.98-1.00)	1.00 (1.00-1.00)
8	(1.02319)*miR-8073+(−1.08749)*miR-6794- 5p+(1.63503)*miR-3196+(−1.02075)*miR-6820- 5p+(0.559982)*miR-744-5p+(0.613777)*miR-4433a- 3p+(−0.277379)*miR-4734+(0.0347834)*miR-7641-17.08907	0.99 (0.98 -1.00)	0.99 (0.98-1.00)	0.99 (0.99-1.00)	1.00 (1.00-1.00)

Abbreviation: AUC, area under the curve.

### Validation of the EC Index

**Figure 3.**
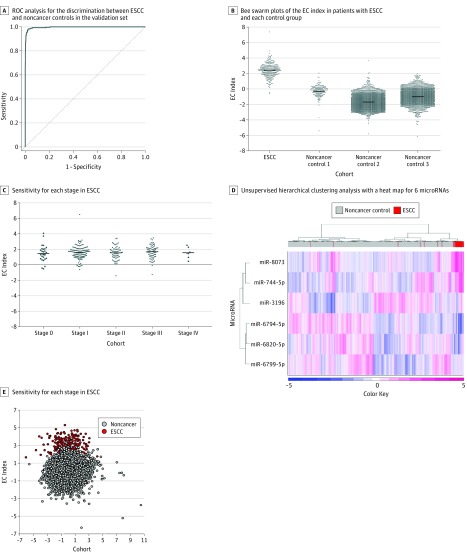
Validation of the EC Index A, The sensitivity, specificity, and area under the curve (AUC) were 0.96 (95% CI, 0.93-0.98), 0.98 (95% CI, 0.98-0.99), and 1.00 (95% CI, 1.00-1.00), respectively. B, The sensitivity for esophageal squamous cell carcinoma (ESCC) was 0.96. The specificities for noncancer control 1, noncancer control 2, and noncancer control 3 were 0.95, 0.99, and 0.97, respectively. C, The sensitivities for stage 0, stage I, stage II, stage III, and stage IV were 0.89, 0.95, 0.98, 0.97, and 1.00, respectively. D, The ESCC and noncancer control samples in the validation set were plotted. Each microRNA expression was standardized by considering the mean as 0 and the SD as 1 in all features, which gives all microRNAs equal weight. E, The axes show the first 2 principal components. The first 2 principal components explain 57.3% of variance. The fractions of explained variance for the first principal component and the second principal component were 33.7% and 23.6%, respectively. ROC indicates receiver operating characteristic curve.

In the validation cohort, the area under the receiver operating curve for the EC index was 1.00, with sensitivity of 0.96 and specificity of 0.98 (Figure 3A). Figure 3B shows bee swarm plots of the EC index in patients with ESCC and each control group. The specificity was 0.95 to 0.99 in each control group. The sensitivity by stage was 0.89 for stage 0, 0.95 for stage I, 0.98 for stage II, 0.97 for stage III, and 1.00 for stage IV (Figure 3C). Unsupervised hierarchical clustering analysis with a heat map and principal component analysis showed that the 6 miRNAs effectively differentiated ESCC from noncancer controls (Figure 3D and E). Multivariable logistic regression analyses were performed that included age, sex, and the EC index, and the EC index was independently associated with the presence of ESCC (odds ratio, 38.2; 95% CI, 23.1-63.3) (eTable 2 in the Supplement).

## Discussion

Studies^10,11^ analyzing serum miRNAs as diagnostic markers show that the expression of serum miRNAs is related to ESCC. Zhang et al^10^ tested the serum samples of 290 patients with ESCC and 140 controls using sequencing technology for an initial screen of miRNAs and a hydrolysis probe–based stem-loop quantitative reverse transcription–polymerase chain reaction assay of training and validation sets. The results showed that 7 miRNAs (miR-10a, miR-22, miR-100, miR-148b, miR-223, miR-133a, and miR-127-3p) were significantly upregulated in the serum of patients with ESCC compared with the control patients (the AUC for the 7 miRNAs ranged from 0.817 to 0.949). Takeshita et al^11^ tested serum samples from 101 patients with ESCC and 46 control patients in a miRNA array and found that serum miR-1246 was upregulated in ESCC, with an AUC of 0.754, a sensitivity of 0.713, and a specificity of 0.739 for distinguishing patients with ESCC from control patients. The present study included 566 patients with ESCC and 4965 control patients, which is the largest-scale study for designing a diagnostic model of ESCC reported to date, to our knowledge. We developed a diagnostic model of serum miRNAs (named the EC index) that achieved high sensitivity and specificity (0.96 and 0.98, respectively, in the validation cohort).

To our knowledge, no population-based screening test has been established for ESCC to date; therefore, the EC index is a promising candidate as a screening tool before endoscopy. A study^12^ showed that the sensitivity and specificity for detecting colorectal cancer by stool sample DNA testing were 0.923 and 0.866, respectively, whereas those for a fecal sample immunochemical test were 0.738 and 0.949, respectively. These tests are widely used as population screening methods for colorectal cancer. The EC index achieved a higher sensitivity and specificity than these methods. Furthermore, the EC index showed high sensitivity not only in patients with advanced disease but also in those with early ESCC (0.89 for stage 0 and 0.95 for stage I), who may be candidates for curative treatment.

Because the primary role of a screening test is to detect diseases, sensitivity is an important determinant of the usefulness of a screening test. It is estimated that a negative result in the EC index reduces the risk of ESCC from a baseline of approximately 0.031% (31 in 100 000) to 0.0013% (1 minus negative predictive value) in Japanese men and from approximately 0.0056% (5.6 in 100 000) to 0.00023% in Japanese women.^2^ A positive result in the EC index may increase the probability of ESCC from a baseline risk of 0.031% to 1.5% (positive predictive value) in Japanese men and from 0.0056% to 0.27% in Japanese women. Specificity is another important measure in a cancer screening test. High specificity can reduce the possibility of false-positive results, which require additional tests, such as esophagogastroduodenoscopy, after screening. However, most individuals with a positive EC index result may not have ESCC because of the low prevalence of ESCC. Another potential use of the EC index is for surveillance after treatment, and further study is needed to confirm the utility of the EC index as a surveillance tool for recurrence or disease progression. The project from our group aims to develop diagnostic models not only for ESCC but also for other cancers, including gastric, lung, hepatic, biliary tract, pancreatic, colon, ovarian, prostate, bladder, breast, sarcoma, and glioma.^7,13^ Many countries are making efforts to increase the rate of participation in cancer screening programs to improve early-stage detection and provide appropriate treatment. Less invasive tests may help increase the number of participants in screening programs. The development of a highly accurate diagnostic index for multiple cancers using serum miRNAs would facilitate multicancer screening using a small amount of blood, and such an index would thus be a useful modality. However, obtaining evidence of the usefulness of a serum miRNA test for population-based screening is difficult in ESCC because of the low prevalence of the disease. In addition, the selection of individuals eligible for multicancer screening assays and the cancer types that should be screened must be considered on a regional basis because of regional differences in cancer prevalence.

The EC index includes 6 miRNAs (miR-8073, miR-6820-5p, miR-6794-5p, miR-3196, miR-744-5p, and miR-6799-5p). The serum expression of miR-8073, miR-3196, and miR-744-5p was higher in patients with ESCC than in control patients, and these miRNAs may be candidate oncogenic miRNAs. In contrast, the expression of miR-6820-5p, miR-6794-5p, and miR-6799-5p was lower in patients with ESCC than in control patients, suggesting that these are tumor suppressor miRNAs. Although the roles of these miRNAs in carcinogenesis remain unclear, the cancer-related functions of these miRNAs were suggested previously. Cui et al^14^ developed a diagnostic model for breast cancer using 3 miRNAs, including miR-8073, although the role of miR-8073 in carcinogenesis is unknown. Another study^15^ showed that miR-3196 inhibits apoptosis in lung cancer cells by targeting the p53-upregulated modulator of apoptosis. Because somatic mutation of p53 is frequently detected in patients with ESCC, the molecular mechanism underlying the influence of miR-3196 may be similar between ESCC and lung cancer. The upregulation of miR-6794-5p was suggested to underlie the proliferation of triple-negative breast cancer cells.^16^ Furthermore, miR-744-5p is downregulated in patients with primary lung cancer showing resistance to epidermal growth factor receptor tyrosine kinase inhibitors.^17^ In addition, miR-6799-5p expression is lower in the serum of patients with pancreatic cancer than in that of healthy controls^18^; therefore, it is considered a tumor suppressor miRNA, consistent with the results of the present study. We were unable to find any literature on the role of miR-6820-5p in carcinogenesis.

### Limitations

Our study has several limitations. The study was a retrospective analysis using archival samples, and an external validation cohort for patients with ESCC was not available. Variation in sample collection and storage may have influenced the EC index because samples were collected from different institutions. To confirm that the EC index can distinguish not only external controls but also internal controls, we included patients with benign diseases from the NCCH whose serum samples were collected and stored under the same conditions as those from patients with ESCC. To confirm the usefulness of the EC index for detecting ESCC in a large cohort, we used the low incidence rate of ESCC as a means to set the number of patients with ESCC vs control patients without cancer in the validation data set. The EC index achieved high specificity in the NCCH benign cohort (0.95) and other control groups. Although the multivariable analysis included age and sex, it did not include other known risk factors for ESCC, such as smoking and drinking status, which is a limitation of the study. The EC index achieved high accuracy for discriminating between patients with ESCC and control patients; however, the risk factors may have influenced circulating miRNAs and the odds ratio in the multivariable analysis. We are conducting a prospective study by collecting serum samples from multiple institutions to overcome these limitations.

## Conclusions

In this study, we developed what may be a serum miRNA discriminant model (named the EC index) for detecting ESCC with high sensitivity and specificity regardless of stage. A prospective study that includes multiple institutions is underway to confirm our observations.

## Notes

Supplement. **eMethods.** Algorithm: Combinatorial Optimization for Multicandidate miRNAs **eTable 1.** Top 20 Models for Each Number of miRNAs Included in the Training Set **eTable 2.** Independent Association Between the Six-miRNA Panel (EC Index) and the Presence of Esophageal Squamous Cell Carcinoma **eFigure 1.** Dot Plots for Six miRNAs in ESCC and Non-cancer Controls in the Training Set **eFigure 2.** ROC Analysis of Individual miRNAs for Distinguishing ESCC From Non-cancer Controls in the Training Set
