# Incorrect X-Axis, Y-Axis, and Panel Labels

**DOI:** 10.1001/jamanetworkopen.2019.7401

**Published:** 2019-06-28

In the Original Investigation titled “Development and Validation of an Esophageal Squamous Cell Carcinoma Detection Model by Large-Scale MicroRNA Profiling,”^1^ published May 24, 2019, the labels for the x-axis, y-axis, and panel for Figure 3E were incorrect. The x-axis now reads “Principal Component 1”; y-axis, “Principal Component 2”; and panel label, “Principal component analysis.” This article has been corrected.^1^
