# Supplementary material for: Development and Validation of an Esophageal Squamous Cell Carcinoma Detection Model by Large-Scale MicroRNA Profiling
Source: JAMA Netw Open. 2019 May 24;2(5):e194573. doi: 10.1001/jamanetworkopen.2019.4573 (PMC6632131; doi:10.1001/jamanetworkopen.2019.4573)
Supplement: Supplement. — eMethods. Algorithm: Combinatorial Optimization for Multicandidate miRNAs eTable 1. Top 20 Models for Each Number of miRNAs Included in the Training Set eTable 2. Independent Association Between the Six-miRNA Panel (EC Index) and the Presence of Esophageal Squamous Cell Carcinoma eFigure 1. Dot Plots for Six miRNAs in ESCC and Non-cancer Controls in the Training Set eFigure 2. ROC Analysis of Individual miRNAs for Distinguishing ESCC From Non-cancer Controls in the Training Set [file jamanetwopen-2-e194573-s001.pdf]

Supplementary Online Content

Sudo K, Kato K, Matsuzaki J, et al. Development and validation of an esophageal squamous cell carcinoma detection model by large-scale microRNA profiling. *JAMA Netw Open*. 2019;2(5):e194573. doi:10.1001/jamanetworkopen.2019.4573

eMethods. Algorithm: Combinatorial Optimization for Multicandidate miRNAs

eTable 1. Top 20 Models for Each Number of miRNAs Included in the Training Set

eTable 2. Independent Association Between the Six-miRNA Panel (EC Index) and the Presence of Esophageal Squamous Cell Carcinoma

eFigure 1. Dot Plots for Six miRNAs in ESCC and Non-cancer Controls in the Training Set

eFigure 2. ROC Analysis of Individual miRNAs for Distinguishing ESCC From Non-cancer Controls in the Training Set

This supplementary material has been provided by the authors to give readers additional information about their work.

© 2019 Sudo K et al. *JAMA Network Open.*

eMethods. Algorithm: Combinatorial Optimization for Multicandidate miRNAs

Notation:

N: Number of candidate sets of miRNAs.

M: Maximum combined number of miRNAs in each candidate set.

T: Total number of miRNA.

Score: Accuracy = (TP + TN) / (TP + FP + FN + TN), where the meaning of each variable is as follows:

	Cancer	No cancer
Prediction positive	TP: number of true positives	FP: number of false positives
Prediction negative	FN: number of false negatives	TN: number of true negatives

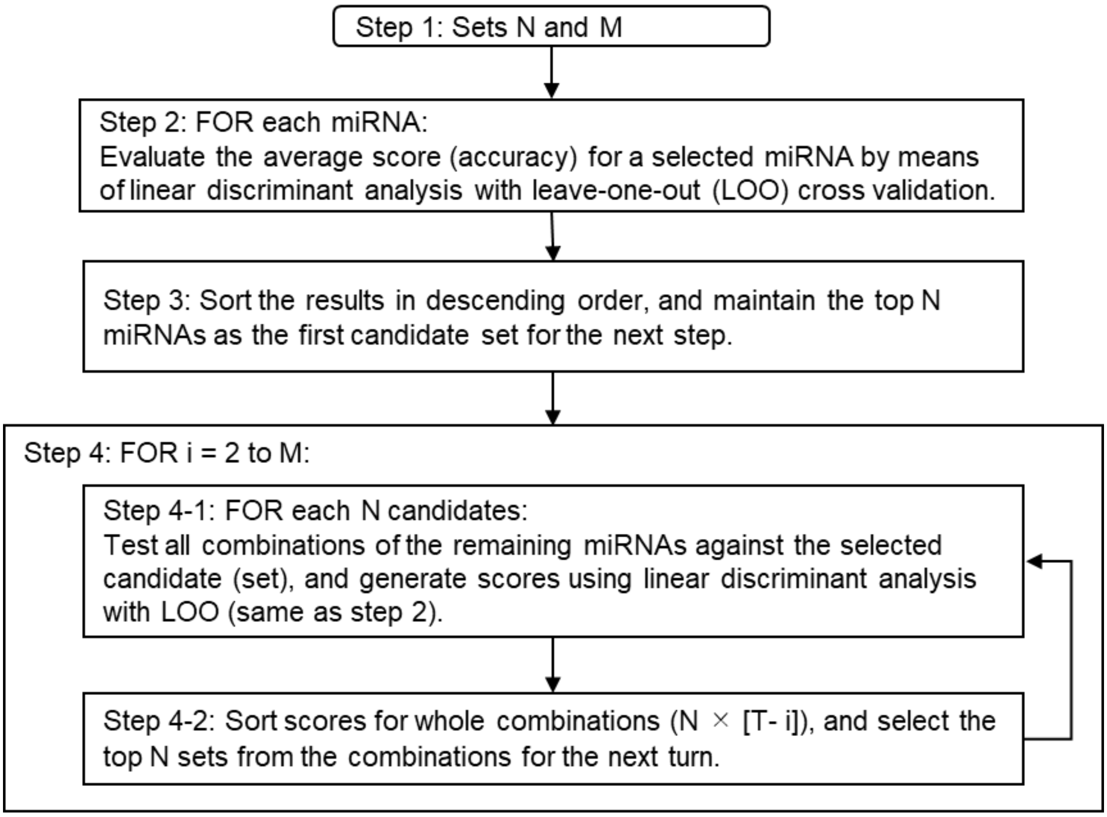

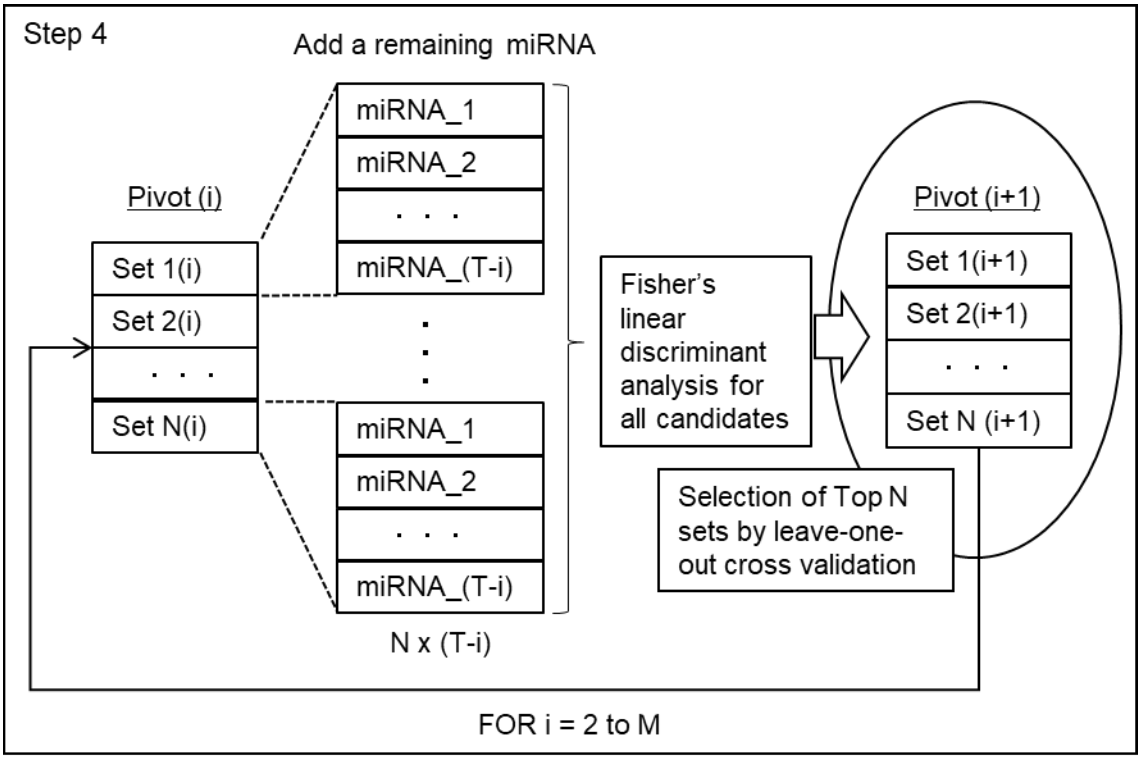

© 2019 Sudo K et al. *JAMA Network Open*.

eTable 1. Top 20 Models for Each Number of miRNAs Included in the Training Set

Number of miRNAs	Model candidates	Sensitivity	Specificity	Accuracy	Area under the curve
1	(-1.81483)*hsa-miR-6800-5p+14.33326	0.90	0.76	0.83	0.90
1	(1.15286)*hsa-miR-8073-8.0801003	0.89	0.74	0.81	0.87
1	(-0.810638)*hsa-miR-8071+5.15911	0.79	0.85	0.82	0.90
1	(-1.36986)*hsa-miR-7111-5p+9.30618	0.70	0.90	0.80	0.86
1	(0.773562)*hsa-miR-320b-4.33607	0.88	0.71	0.79	0.87
1	(-0.962817)*hsa-miR-6763-5p+6.03093	0.66	0.94	0.80	0.84
1	(-0.784622)*hsa-miR-7845-5p+4.9253	0.81	0.82	0.81	0.88
1	(1.06361)*hsa-miR-4258-9.9989	0.9399	0.6749	0.8074	0.8355
1	(2.34844)*hsa-miR-6789-5p-22.83756	0.8375	0.7067	0.7721	0.8186
1	(-1.35374)*hsa-miR-6785-5p+10.4532	0.6926	0.8481	0.7703	0.839
1	(1.33748)*hsa-miR-663b-11.4333	0.841	0.6926	0.7668	0.802
1	(-2.63879)*hsa-miR-6794-5p+20.7041	0.6996	0.8622	0.7809	0.8461
1	(-2.10977)*hsa-miR-711+16.39257	0.7279	0.7951	0.7615	0.8063
1	(-0.754848)*hsa-miR-4667-5p+4.49839	0.8021	0.7491	0.7756	0.8482
1	(3.74734)*hsa-miR-4417-30.9434	0.8869	0.6396	0.7633	0.8322
1	(1.80442)*hsa-miR-1225-	0.9399	0.6184	0.7792	0.8126

© 2019 Sudo K et al. *JAMA Network Open.*

	3p-11.8447				
1	(-3.27384)*hsa-miR-1268b+31.8244	0.8198	0.7032	0.7615	0.8284
1	(-0.866631)*hsa-miR-7110-5p+5.8372	0.8021	0.7385	0.7703	0.8464
1	(0.850166)*hsa-miR-4532-11.1332	0.8021	0.6961	0.7491	0.8238
1	(-2.74092)*hsa-miR-6765-5p+28.763	0.8975	0.629	0.7633	0.7978
2	(1.02272)*hsa-miR-8073+(-2.17698)*hsa-miR-6794-5p+10.0051	0.9011	0.9435	0.9223	0.9763
2	(0.64388)*hsa-miR-320b+(-2.13999)*hsa-miR-6794-5p+13.2985	0.8763	0.9399	0.9081	0.9589
2	(-0.564438)*hsa-miR-8071+(0.540159)*hsa-miR-320b+0.514926	0.9187	0.8657	0.8922	0.9511
2	(0.627784)*hsa-miR-320b+(-0.50154)*hsa-miR-4667-5p-0.77292	0.8799	0.9187	0.8993	0.9502
2	(1.04968)*hsa-miR-4258+(-1.4109)*hsa-miR-6820-5p+0.0145221	0.9435	0.8339	0.8887	0.9385
2	(1.20557)*hsa-miR-8073+(-1.23813)*hsa-miR-6741-5p+0.352889	0.8869	0.9081	0.8975	0.9631
2	(-0.570414)*hsa-miR-8071+(0.74962)*hsa-miR-4258-3.7977	0.9329	0.8375	0.8852	0.9321
2	(-1.78575)*hsa-miR-6800-5p+(0.600954)*hsa-miR-1908-3p+10.09235	0.9399	0.8375	0.8887	0.9415
2	(-1.25927)*hsa-miR-6800-5p+(-0.457934)*hsa-miR-	0.8975	0.8763	0.8869	0.9467

© 2019 Sudo K et al. *JAMA Network Open.*

	8071+12.78498				
2	(-2.24853)*hsa-miR-6794-5p+(0.774277)*hsa-miR-4454+9.59826	0.8339	0.9611	0.8975	0.9482
2	(0.642089)*hsa-miR-320b+(-0.664931)*hsa-miR-6763-5p+0.793563	0.9152	0.8516	0.8834	0.95
2	(0.842483)*hsa-miR-4258+(-2.02832)*hsa-miR-6794-5p+7.89936	0.9293	0.8375	0.8834	0.9327
2	(1.87553)*hsa-miR-6789-5p+(-1.18314)*hsa-miR-6785-5p-9.056248	0.9046	0.8587	0.8816	0.9487
2	(0.599102)*hsa-miR-320b+(-0.519549)*hsa-miR-7845-5p-0.297385	0.8869	0.8799	0.8834	0.9411
2	(-0.639626)*hsa-miR-6763-5p+(0.86519)*hsa-miR-4258-4.38933	0.9505	0.8127	0.8816	0.9132
2	(-2.33496)*hsa-miR-6794-5p+(0.955668)*hsa-miR-1260b+9.7683	0.8339	0.9364	0.8852	0.943
2	(-1.46029)*hsa-miR-6800-5p+(-0.48008)*hsa-miR-6763-5p+14.58889	0.8869	0.8728	0.8799	0.9365
2	(0.80359)*hsa-miR-320b+(-1.547)*hsa-miR-3141+7.24262	0.8728	0.9081	0.8905	0.9372
2	(0.821622)*hsa-miR-8073+(-0.510951)*hsa-miR-8071-2.60716	0.9435	0.8233	0.8834	0.9436
2	(-1.40636)*hsa-miR-6800-5p+(-0.405728)*hsa-miR-7845-5p+13.6617	0.9505	0.8092	0.8799	0.9401

© 2019 Sudo K et al. *JAMA Network Open.*

3	(1.09219)*hsa-miR-8073+(-2.28576)*hsa-miR-6794-5p+(0.826147)*hsa-miR-3180-3p+3.06847	0.947	0.9541	0.9505	0.9811
3	(1.16857)*hsa-miR-8073+(-2.21428)*hsa-miR-6794-5p+(-0.335046)*hsa-miR-4734+13.64675	0.9576	0.9435	0.9505	0.9823
3	(0.998129)*hsa-miR-8073+(-2.24785)*hsa-miR-6794-5p+(0.653115)*hsa-miR-4665-5p+4.72072	0.9293	0.9823	0.9558	0.9805
3	(1.08151)*hsa-miR-8073+(-1.93149)*hsa-miR-6794-5p+(1.10605)*hsa-miR-3196-5.57101	0.9576	0.947	0.9523	0.9839
3	(0.784039)*hsa-miR-8073+(-2.08019)*hsa-miR-6794-5p+(0.471349)*hsa-miR-4258+6.408017	0.9611	0.9293	0.9452	0.9815
3	(1.06216)*hsa-miR-8073+(-2.06836)*hsa-miR-6794-5p+(0.345174)*hsa-miR-1915-3p+5.3698498	0.9435	0.9399	0.9417	0.9805
3	(0.729225)*hsa-miR-8073+(-2.15169)*hsa-miR-6794-5p+(0.348516)*hsa-miR-320b+9.97807	0.9505	0.9364	0.9435	0.9816

© 2019 Sudo K et al. *JAMA Network Open.*

3	(0.87809)*hsa-miR-8073+(-2.28411)*hsa-miR-6794-5p+(0.483014)*hsa-miR-4447+8.70221	0.9329	0.9682	0.9505	0.9844
3	(1.08098)*hsa-miR-8073+(-2.18677)*hsa-miR-6794-5p+(0.380537)*hsa-miR-1343-5p+5.98441	0.947	0.9435	0.9452	0.9795
3	(0.840857)*hsa-miR-8073+(-1.83683)*hsa-miR-6794-5p+(-0.701582)*hsa-miR-6800-5p+14.0709	0.9364	0.9576	0.947	0.9838
3	(1.09843)*hsa-miR-8073+(-1.72704)*hsa-miR-6794-5p+(-0.56324)*hsa-miR-6741-5p+10.145324	0.947	0.9293	0.9382	0.9839
3	(1.12116)*hsa-miR-8073+(-2.13956)*hsa-miR-6794-5p+(-0.325315)*hsa-miR-4758-5p+12.054895	0.9541	0.9258	0.9399	0.9801
3	(-0.566497)*hsa-miR-8071+(0.597159)*hsa-miR-320b+(0.836653)*hsa-miR-1915-3p-8.50582	0.9647	0.9117	0.9382	0.9676
3	(-0.547104)*hsa-miR-8071+(0.551361)*hsa-miR-320b+(1.5049)*hsa-miR-3196-17.810646	0.9682	0.9046	0.9364	0.9689
3	(1.10157)*hsa-miR-8073+(-2.17152)*hsa-	0.9576	0.9187	0.9382	0.9785

© 2019 Sudo K et al. *JAMA Network Open.*

	miR-6794-5p+(-0.784758)*hsa-miR-1227-5p+17.23629				
3	(1.0154)*hsa-miR-8073+(-2.08664)*hsa-miR-6794-5p+(0.362195)*hsa-miR-4497+4.90523	0.9435	0.9329	0.9382	0.9788
3	(0.938174)*hsa-miR-8073+(-2.13256)*hsa-miR-6794-5p+(0.185607)*hsa-miR-6872-3p+9.3243	0.947	0.9364	0.9417	0.9786
3	(1.03451)*hsa-miR-8073+(-2.01704)*hsa-miR-6794-5p+(-0.488938)*hsa-miR-3141+12.60459	0.9541	0.9187	0.9364	0.9803
3	(0.971456)*hsa-miR-8073+(-2.27072)*hsa-miR-6794-5p+(0.235129)*hsa-miR-6826-5p+9.58059	0.9505	0.9293	0.9399	0.9781
3	(0.851687)*hsa-miR-8073+(-2.39374)*hsa-miR-6794-5p+(0.362871)*hsa-miR-296-3p+10.531	0.947	0.9364	0.9417	0.982
4	(0.996309)*hsa-miR-8073+(-1.47812)*hsa-miR-6794-5p+(1.20479)*hsa-miR-4665-5p+(-1.08956)*hsa-miR-6820-5p+1.83975	0.9611	0.9682	0.9647	0.9941
4	(0.905996)*hsa-miR-8073+(-1.84995)*hsa-	0.9611	0.9717	0.9664	0.9927

© 2019 Sudo K et al. *JAMA Network Open.*

	miR-6794-5p+(-0.784975)*hsa-miR-6741-5p+(0.483014)*hsa-miR-296-3p+10.77927				
4	(0.923188)*hsa-miR-8073+(-1.60671)*hsa-miR-6794-5p+(-0.570465)*hsa-miR-6741-5p+(0.768437)*hsa-miR-1225-3p+5.16969	0.9647	0.9647	0.9647	0.9908
4	(1.15762)*hsa-miR-8073+(-1.68941)*hsa-miR-6794-5p+(1.48877)*hsa-miR-3180-3p+(-0.882079)*hsa-miR-6820-5p-1.39668	0.9682	0.9576	0.9629	0.9915
4	(1.11421)*hsa-miR-8073+(-1.23835)*hsa-miR-6794-5p+(1.54811)*hsa-miR-3196+(-0.769775)*hsa-miR-6820-5p-10.96138	0.9753	0.9576	0.9664	0.9937
4	(1.12311)*hsa-miR-8073+(-1.98329)*hsa-miR-6794-5p+(1.00853)*hsa-miR-3180-3p+(-0.387524)*hsa-miR-4688+1.60299	0.9647	0.9611	0.9629	0.9888
4	(0.946274)*hsa-miR-8073+(-2.37044)*hsa-miR-6794-5p+(0.675578)*hsa-miR-3180-3p+(0.451778)*hsa-miR-4447+3.22476	0.9576	0.9647	0.9611	0.9871

© 2019 Sudo K et al. *JAMA Network Open.*

4	(0.906951)*hsa-miR-8073+(-1.6235)*hsa-miR-6794-5p+(1.05146)*hsa-miR-3196+(-0.676705)*hsa-miR-6800-5p-0.859582	0.9682	0.9541	0.9611	0.9902
4	(1.15813)*hsa-miR-8073+(-1.63862)*hsa-miR-6794-5p+(1.20275)*hsa-miR-3196+(-0.270151)*hsa-miR-1247-3p-7.85461	0.9682	0.9505	0.9594	0.9876
4	(1.11107)*hsa-miR-8073+(-1.54096)*hsa-miR-6794-5p+(1.32566)*hsa-miR-3196+(-0.409291)*hsa-miR-4688-8.69495	0.9541	0.9647	0.9594	0.9918
4	(0.942331)*hsa-miR-8073+(-2.05375)*hsa-miR-6794-5p+(1.0717)*hsa-miR-3196+(0.473737)*hsa-miR-4447-6.40388	0.9576	0.9717	0.9647	0.9899
4	(0.886557)*hsa-miR-8073+(-1.40602)*hsa-miR-6794-5p+(1.97685)*hsa-miR-3196+(-1.34802)*hsa-miR-6781-5p-5.76677	0.9717	0.9541	0.9629	0.9886
4	(0.737028)*hsa-miR-8073+(-1.62)*hsa-miR-6794-5p+(0.621026)*hsa-miR-4258+(-0.528752)*hsa-miR-	0.9399	0.9859	0.9629	0.9905

© 2019 Sudo K et al. *JAMA Network Open.*

	4688+4.97869				
4	(0.829691)*hsa-miR-8073+(-2.2894)*hsa-miR-6794-5p+(0.451002)*hsa-miR-4447+(0.272308)*hsa-miR-940+7.44503	0.9647	0.9576	0.9611	0.9862
4	(1.04594)*hsa-miR-8073+(-1.98493)*hsa-miR-6794-5p+(0.823438)*hsa-miR-3180-3p+(-0.221895)*hsa-miR-4667-5p+2.3517	0.9541	0.9647	0.9594	0.988
4	(1.27001)*hsa-miR-8073+(-1.73651)*hsa-miR-6794-5p+(-0.376944)*hsa-miR-4734+(-0.607883)*hsa-miR-6741-5p+14.146891	0.9647	0.9541	0.9594	0.9908
4	(1.05514)*hsa-miR-8073+(-2.14814)*hsa-miR-6794-5p+(0.768452)*hsa-miR-4665-5p+(-0.121096)*hsa-miR-7641+3.36584	0.9576	0.9541	0.9558	0.9831
4	(1.02854)*hsa-miR-8073+(-1.73682)*hsa-miR-6794-5p+(1.75583)*hsa-miR-3196+(0.771538)*hsa-miR-4433a-3p-20.6066	0.9435	0.9753	0.9594	0.9878
4	(0.961816)*hsa-miR-8073+(-1.57103)*hsa-miR-6794-	0.9753	0.9505	0.9629	0.9898

© 2019 Sudo K et al. *JAMA Network Open.*

	5p+(1.19817)*hsa-miR-3196+(-0.280658)*hsa-miR-8071-6.89972				
4	(0.727813)*hsa-miR-8073+(-1.44141)*hsa-miR-6794-5p+(0.608807)*hsa-miR-4258+(-0.80038)*hsa-miR-6820-5p+6.2459	0.9717	0.9435	0.9576	0.9893
5	(0.9634)*hsa-miR-8073+(-1.07335)*hsa-miR-6794-5p+(1.36201)*hsa-miR-3196+(-1.06632)*hsa-miR-6820-5p+(0.576916)*hsa-miR-744-5p-11.99418	0.9894	0.9753	0.9823	0.9972
5	(0.994866)*hsa-miR-8073+(-1.08646)*hsa-miR-6794-5p+(1.40659)*hsa-miR-3196+(-1.08533)*hsa-miR-6820-5p+(0.585181)*hsa-miR-6132-12.9304	0.9753	0.9859	0.9806	0.9969
5	(0.930098)*hsa-miR-8073+(-1.67591)*hsa-miR-6794-5p+(-0.845495)*hsa-miR-6741-5p+(0.476519)*hsa-miR-296-3p+(0.327762)*hsa-miR-187-5p+7.16995	0.9823	0.9717	0.977	0.9941
5	(0.968777)*hsa-miR-8073+(-1.21593)*hsa-miR-6794-5p+(1.38546)*hsa-miR-	0.9788	0.9823	0.9806	0.9972

© 2019 Sudo K et al. *JAMA Network Open*.

	3196+(-1.12763)*hsa-miR-6820-5p+(0.731225)*hsa-miR-4505-12.75564				
5	(0.837163)*hsa-miR-8073+(-2.03545)*hsa-miR-6794-5p+(0.933449)*hsa-miR-3180-3p+(0.442757)*hsa-miR-4447+(-0.310599)*hsa-miR-8071+1.08754	0.9753	0.9788	0.977	0.9941
5	(0.909498)*hsa-miR-8073+(-1.46977)*hsa-miR-6794-5p+(1.02824)*hsa-miR-4665-5p+(-1.26326)*hsa-miR-6820-5p+(0.491504)*hsa-miR-4505+0.466531	0.9788	0.9753	0.977	0.9962
5	(0.897001)*hsa-miR-8073+(-1.3566)*hsa-miR-6794-5p+(1.0083)*hsa-miR-4665-5p+(-1.24052)*hsa-miR-6820-5p+(0.432073)*hsa-miR-744-5p+0.650182	0.9859	0.9647	0.9753	0.9964
5	(0.93483)*hsa-miR-8073+(-1.86519)*hsa-miR-6794-5p+(-0.768271)*hsa-miR-6741-5p+(0.385744)*hsa-miR-296-3p+(0.484688)*hsa-miR-4665-5p+6.84966	0.9717	0.9788	0.9753	0.9927

© 2019 Sudo K et al. *JAMA Network Open.*

5	(1.00518)*hsa-miR-8073+(-1.17321)*hsa-miR-6794-5p+(1.33167)*hsa-miR-3196+(-1.00317)*hsa-miR-6820-5p+(0.650778)*hsa-miR-6780b-5p-13.15327	0.9894	0.9682	0.9788	0.9964
5	(0.894453)*hsa-miR-8073+(-1.07906)*hsa-miR-6794-5p+(1.00193)*hsa-miR-3196+(-0.982991)*hsa-miR-6820-5p+(0.872142)*hsa-miR-1225-3p-8.7791	0.9717	0.9823	0.977	0.9965
5	(1.06262)*hsa-miR-8073+(-1.24905)*hsa-miR-6794-5p+(1.43156)*hsa-miR-3196+(-1.04137)*hsa-miR-6820-5p+(0.668557)*hsa-miR-4327-13.38145	0.9859	0.9717	0.9788	0.9963
5	(0.934089)*hsa-miR-8073+(-1.42546)*hsa-miR-6794-5p+(1.02905)*hsa-miR-4665-5p+(-1.19261)*hsa-miR-6820-5p+(0.45248)*hsa-miR-6780b-5p-0.586233	0.9753	0.9823	0.9788	0.9958
5	(0.880049)*hsa-miR-8073+(-1.31008)*hsa-miR-6794-	0.9788	0.9753	0.977	0.9952

© 2019 Sudo K et al. *JAMA Network Open*.

	5p+(1.09287)*hsa-miR-4665-5p+(-1.00444)*hsa-miR-6820-5p+(-0.486834)*hsa-miR-6800-5p+5.461				
5	(0.922107)*hsa-miR-8073+(-1.37809)*hsa-miR-6794-5p+(1.03868)*hsa-miR-4665-5p+(-1.25009)*hsa-miR-6820-5p+(0.414883)*hsa-miR-6132+0.351125	0.9753	0.9788	0.977	0.9963
5	(0.977985)*hsa-miR-8073+(-1.88407)*hsa-miR-6794-5p+(-0.789987)*hsa-miR-6741-5p+(0.515231)*hsa-miR-296-3p+(0.550468)*hsa-miR-1343-5p+4.86332	0.9717	0.9788	0.9753	0.9939
5	(0.999764)*hsa-miR-8073+(-1.47731)*hsa-miR-6794-5p+(1.26051)*hsa-miR-3180-3p+(-1.14414)*hsa-miR-6820-5p+(0.562809)*hsa-miR-744-5p-3.060296	0.9859	0.9682	0.977	0.9959
5	(0.984242)*hsa-miR-8073+(-1.23924)*hsa-miR-6794-5p+(1.50895)*hsa-miR-3196+(-0.647528)*hsa-miR-6820-5p+(1.20021)*hsa-miR-	0.9788	0.9647	0.9717	0.9937

© 2019 Sudo K et al. *JAMA Network Open.*

	4417-20.56153				
5	(0.986583)*hsa-miR-8073+(-1.29292)*hsa-miR-6794-5p+(1.15814)*hsa-miR-3196+(-0.704269)*hsa-miR-6800-5p+(-0.297347)*hsa-miR-1247-3p-3.15785	0.9859	0.9611	0.9735	0.9936
5	(0.96789)*hsa-miR-8073+(-1.45414)*hsa-miR-6794-5p+(1.33949)*hsa-miR-3196+(-0.371061)*hsa-miR-4688+(1.34619)*hsa-miR-4417-20.0059	0.9823	0.9611	0.9717	0.9939
5	(1.04692)*hsa-miR-8073+(-1.28378)*hsa-miR-6794-5p+(1.17152)*hsa-miR-4665-5p+(-0.985668)*hsa-miR-6820-5p+(-0.349072)*hsa-miR-6741-5p+1.968637	0.9647	0.9788	0.9717	0.9957
6	(0.961037)*hsa-miR-8073+(-0.962054)*hsa-miR-6794-5p+(1.31647)*hsa-miR-3196+(-1.0132)*hsa-miR-6820-5p+(0.657628)*hsa-miR-744-5p+(-0.406723)*hsa-miR-6799-5p-9.799262	0.9965	0.9788	0.9876	0.998

© 2019 Sudo K et al. *JAMA Network Open.*

6	(0.929103)*hsa-miR-8073+(-1.03973)*hsa-miR-6794-5p+(1.78475)*hsa-miR-3196+(-0.952522)*hsa-miR-6820-5p+(0.561485)*hsa-miR-744-5p+(0.562424)*hsa-miR-4433a-3p-22.05395	0.9894	0.9823	0.9859	0.9987
6	(0.918953)*hsa-miR-8073+(-0.96918)*hsa-miR-6794-5p+(1.35085)*hsa-miR-3196+(-1.01209)*hsa-miR-6820-5p+(0.908879)*hsa-miR-744-5p+(-0.552962)*hsa-miR-4327-10.61214	0.9929	0.9788	0.9859	0.9971
6	(0.8796)*hsa-miR-8073+(-1.23586)*hsa-miR-6794-5p+(1.35713)*hsa-miR-3196+(-0.998032)*hsa-miR-6820-5p+(0.490403)*hsa-miR-744-5p+(0.389081)*hsa-miR-4447-12.29229	0.9929	0.9823	0.9876	0.9985
6	(1.07283)*hsa-miR-8073+(-1.1885)*hsa-miR-6794-5p+(1.33128)*hsa-miR-3180-3p+(-0.881748)*hsa-miR-6820-5p+(1.08909)*hsa-miR-744-5p+(-0.833805)*hsa-miR-6511a-5p-7.024127	0.9859	0.9859	0.9859	0.9983

© 2019 Sudo K et al. *JAMA Network Open*.

6	(0.99209)*hsa-miR-8073+(-1.06486)*hsa-miR-6794-5p+(1.49269)*hsa-miR-3196+(-1.0712)*hsa-miR-6820-5p+(0.604199)*hsa-miR-744-5p+(-0.22182)*hsa-miR-4281-11.50377	0.9894	0.9753	0.9823	0.9978
6	(0.981803)*hsa-miR-8073+(-1.11214)*hsa-miR-6794-5p+(1.36272)*hsa-miR-3196+(-1.03897)*hsa-miR-6820-5p+(0.572765)*hsa-miR-744-5p+(-0.0501423)*hsa-miR-4485-5p-11.668904	0.9894	0.9753	0.9823	0.9973
6	(1.01256)*hsa-miR-8073+(-1.09476)*hsa-miR-6794-5p+(1.55025)*hsa-miR-3196+(-0.984921)*hsa-miR-6820-5p+(0.508605)*hsa-miR-744-5p+(-0.212451)*hsa-miR-486-3p-12.70643	0.9859	0.9823	0.9841	0.9976
6	(0.97358)*hsa-miR-8073+(-1.08593)*hsa-miR-6794-5p+(1.30105)*hsa-miR-3196+(-1.07128)*hsa-miR-6820-5p+(0.585552)*hsa-miR-744-5p+(0.101608)*hsa-miR-1343-5p-12.24641	0.9929	0.9753	0.9841	0.9974

© 2019 Sudo K et al. *JAMA Network Open.*

6	(0.957281)*hsa-miR-8073+(-1.09856)*hsa-miR-6794-5p+(1.3584)*hsa-miR-3196+(-1.09548)*hsa-miR-6820-5p+(0.470978)*hsa-miR-744-5p+(0.170659)*hsa-miR-4505-12.18281	0.9929	0.9753	0.9841	0.9974
6	(0.943159)*hsa-miR-8073+(-1.07123)*hsa-miR-6794-5p+(1.51644)*hsa-miR-3196+(-1.0478)*hsa-miR-6820-5p+(0.567743)*hsa-miR-744-5p+(-0.22879)*hsa-miR-6816-5p-11.42026	0.9894	0.9753	0.9823	0.997
6	(0.984276)*hsa-miR-8073+(-1.00648)*hsa-miR-6794-5p+(1.40093)*hsa-miR-3196+(-1.0147)*hsa-miR-6820-5p+(0.552957)*hsa-miR-744-5p+(-0.168776)*hsa-miR-671-5p-12.1006	0.9965	0.9717	0.9841	0.9978
6	(1.00599)*hsa-miR-8073+(-1.06564)*hsa-miR-6794-5p+(1.52912)*hsa-miR-3196+(-0.946477)*hsa-miR-6820-5p+(0.54169)*hsa-miR-744-5p+(-0.267142)*hsa-	0.9894	0.9823	0.9859	0.9981

© 2019 Sudo K et al. *JAMA Network Open.*

	miR-4486-13.09334				
6	(0.929796)*hsa-miR-8073+(-1.00291)*hsa-miR-6794-5p+(1.33949)*hsa-miR-3196+(-0.916395)*hsa-miR-6820-5p+(0.540583)*hsa-miR-744-5p+(-0.176366)*hsa-miR-7845-5p-11.80468	0.9894	0.9823	0.9859	0.9977
6	(1.00663)*hsa-miR-8073+(-0.956273)*hsa-miR-6794-5p+(1.36461)*hsa-miR-3196+(-0.980697)*hsa-miR-6820-5p+(0.603758)*hsa-miR-744-5p+(-0.186226)*hsa-miR-1247-3p-13.0602	0.9859	0.9859	0.9859	0.998
6	(0.948849)*hsa-miR-8073+(-1.07585)*hsa-miR-6794-5p+(1.33776)*hsa-miR-3196+(-1.05037)*hsa-miR-6820-5p+(0.581636)*hsa-miR-744-5p+(-0.0869183)*hsa-miR-6870-5p-11.01057	0.9965	0.9717	0.9841	0.9974
6	(0.948775)*hsa-miR-8073+(-1.14629)*hsa-miR-6794-5p+(1.42898)*hsa-miR-	0.9894	0.9753	0.9823	0.9973

© 2019 Sudo K et al. *JAMA Network Open.*

	3196+(-1.07952)*hsa-miR-6820-5p+(0.590022)*hsa-miR-744-5p+(0.184261)*hsa-miR-937-5p-13.61514				
6	(0.97791)*hsa-miR-8073+(-1.06394)*hsa-miR-6794-5p+(1.38197)*hsa-miR-3196+(-1.06756)*hsa-miR-6820-5p+(0.610253)*hsa-miR-744-5p+(-0.0728517)*hsa-miR-92b-5p-11.9136	0.9929	0.9753	0.9841	0.9975
6	(0.976984)*hsa-miR-8073+(-1.07999)*hsa-miR-6794-5p+(1.4646)*hsa-miR-3196+(-1.07552)*hsa-miR-6820-5p+(0.583662)*hsa-miR-744-5p+(-0.208259)*hsa-miR-4466-10.57534	0.9894	0.9753	0.9823	0.9976
6	(0.963148)*hsa-miR-8073+(-1.07023)*hsa-miR-6794-5p+(1.37354)*hsa-miR-3196+(-1.0338)*hsa-miR-6820-5p+(0.522632)*hsa-miR-744-5p+(-0.114159)*hsa-miR-5090-11.127016	0.9894	0.9753	0.9823	0.9972

© 2019 Sudo K et al. *JAMA Network Open.*

7	(1.04095)*hsa-miR-8073+(-1.08968)*hsa-miR-6794-5p+(1.63487)*hsa-miR-3196+(-0.973975)*hsa-miR-6820-5p+(0.542712)*hsa-miR-744-5p+(0.607937)*hsa-miR-4433a-3p+(-0.267989)*hsa-miR-4734-17.29468	0.9929	0.9894	0.9912	0.9988
7	(1.02803)*hsa-miR-8073+(-0.869999)*hsa-miR-6794-5p+(1.43316)*hsa-miR-3196+(-0.860765)*hsa-miR-6820-5p+(0.564507)*hsa-miR-744-5p+(-0.176918)*hsa-miR-1247-3p+(-0.211972)*hsa-miR-4688-13.709506	0.9929	0.9894	0.9912	0.9985
7	(0.960539)*hsa-miR-8073+(-0.966873)*hsa-miR-6794-5p+(1.31357)*hsa-miR-3196+(-1.01394)*hsa-miR-6820-5p+(0.656591)*hsa-miR-744-5p+(-0.424336)*hsa-miR-6799-5p+(0.0211592)*hsa-miR-504-3p-9.68752	0.9965	0.9788	0.9876	0.998

© 2019 Sudo K et al. *JAMA Network Open.*

7	(0.959844)*hsa-miR-8073+(-0.954994)*hsa-miR-6794-5p+(1.29985)*hsa-miR-3196+(-1.02353)*hsa-miR-6820-5p+(0.6646)*hsa-miR-744-5p+(-0.400395)*hsa-miR-6799-5p+(0.0384965)*hsa-miR-4707-3p-9.91394	0.9965	0.9788	0.9876	0.9979
7	(0.960464)*hsa-miR-8073+(-0.962802)*hsa-miR-6794-5p+(1.32495)*hsa-miR-3196+(-1.01748)*hsa-miR-6820-5p+(0.660601)*hsa-miR-744-5p+(-0.416262)*hsa-miR-6799-5p+(0.0295314)*hsa-miR-5196-5p-9.98622	0.9965	0.9788	0.9876	0.9982
7	(0.96628)*hsa-miR-8073+(-0.96745)*hsa-miR-6794-5p+(1.31063)*hsa-miR-3196+(-1.02637)*hsa-miR-6820-5p+(0.658965)*hsa-miR-744-5p+(-0.415399)*hsa-miR-6799-5p+(0.0274533)*hsa-miR-3928-3p-9.73883	0.9965	0.9788	0.9876	0.998

© 2019 Sudo K et al. *JAMA Network Open.*

7	(0.953409)*hsa-miR-8073+(-0.969508)*hsa-miR-6794-5p+(1.32041)*hsa-miR-3196+(-1.0248)*hsa-miR-6820-5p+(0.645807)*hsa-miR-744-5p+(-0.398638)*hsa-miR-6799-5p+(0.0336723)*hsa-miR-4690-5p-9.8415	0.9965	0.9788	0.9876	0.9981
7	(0.974615)*hsa-miR-8073+(-0.962286)*hsa-miR-6794-5p+(1.28744)*hsa-miR-3196+(-1.02113)*hsa-miR-6820-5p+(0.671653)*hsa-miR-744-5p+(-0.407723)*hsa-miR-6799-5p+(-0.0353105)*hsa-miR-4675-9.311387	0.9965	0.9788	0.9876	0.998
7	(0.953302)*hsa-miR-8073+(-0.982475)*hsa-miR-6794-5p+(1.30732)*hsa-miR-3196+(-1.01569)*hsa-miR-6820-5p+(0.645157)*hsa-miR-744-5p+(-0.400475)*hsa-miR-6799-5p+(0.0445814)*hsa-miR-4706-9.75114	0.9965	0.9788	0.9876	0.9979
7	(0.966594)*hsa-miR-8073+(-0.948358)*hsa-miR-6794-5p+(1.30443)*hsa-miR-	0.9965	0.9788	0.9876	0.9979

© 2019 Sudo K et al. *JAMA Network Open.*

	3196+(-1.01041)*hsa-miR-6820-5p+(0.664693)*hsa-miR-744-5p+(-0.405261)*hsa-miR-6799-5p+(-0.0321212)*hsa-miR-197-5p-9.638007				
7	(0.968862)*hsa-miR-8073+(-0.960263)*hsa-miR-6794-5p+(1.31759)*hsa-miR-3196+(-1.02587)*hsa-miR-6820-5p+(0.665958)*hsa-miR-744-5p+(-0.41206)*hsa-miR-6799-5p+(0.0160642)*hsa-miR-6825-5p-9.91308	0.9965	0.9788	0.9876	0.9981
7	(0.966981)*hsa-miR-8073+(-0.958953)*hsa-miR-6794-5p+(1.31083)*hsa-miR-3196+(-1.01456)*hsa-miR-6820-5p+(0.664381)*hsa-miR-744-5p+(-0.406606)*hsa-miR-6799-5p+(-0.0155745)*hsa-miR-1233-5p-9.665207	0.9965	0.9788	0.9876	0.998
7	(0.95473)*hsa-miR-8073+(-0.980534)*hsa-miR-6794-5p+(1.32586)*hsa-miR-3196+(-1.01761)*hsa-miR-6820-	0.9965	0.9788	0.9876	0.998

© 2019 Sudo K et al. *JAMA Network Open.*

	5p+(0.657056)*hsa-miR-744-5p+(-0.421884)*hsa-miR-6799-5p+(0.0275597)*hsa-miR-6887-5p-9.73106				
7	(0.961167)*hsa-miR-8073+(-0.961937)*hsa-miR-6794-5p+(1.31654)*hsa-miR-3196+(-1.01324)*hsa-miR-6820-5p+(0.657763)*hsa-miR-744-5p+(-0.406704)*hsa-miR-6799-5p+(-0.000446727)*hsa-miR-4649-5p-9.798228	0.9965	0.9788	0.9876	0.998
7	(0.954967)*hsa-miR-8073+(-0.96098)*hsa-miR-6794-5p+(1.33104)*hsa-miR-3196+(-1.00919)*hsa-miR-6820-5p+(0.650832)*hsa-miR-744-5p+(-0.402557)*hsa-miR-6799-5p+(0.0089314)*hsa-miR-4783-3p-10.013746	0.9965	0.9788	0.9876	0.998
7	(0.961644)*hsa-miR-8073+(-0.9943)*hsa-miR-6794-5p+(1.30985)*hsa-miR-3196+(-1.02579)*hsa-miR-6820-5p+(0.654952)*hsa-miR-744-5p+(-0.412551)*hsa-miR-6799-	0.9965	0.9823	0.9894	0.998

© 2019 Sudo K et al. *JAMA Network Open.*

	5p+(0.0757723)*hsa-miR-6779-5p-9.85875				
7	(0.955355)*hsa-miR-8073+(-0.930164)*hsa-miR-6794-5p+(1.31082)*hsa-miR-3196+(-0.994226)*hsa-miR-6820-5p+(0.657917)*hsa-miR-744-5p+(-0.410825)*hsa-miR-6799-5p+(-0.0318577)*hsa-miR-6763-5p-9.84237	0.9965	0.9788	0.9876	0.9977
7	(0.898961)*hsa-miR-8073+(-1.22795)*hsa-miR-6794-5p+(1.43868)*hsa-miR-3196+(-1.00232)*hsa-miR-6820-5p+(0.508896)*hsa-miR-744-5p+(0.382524)*hsa-miR-4447+(-0.138052)*hsa-miR-4281-12.041735	0.9894	0.9859	0.9876	0.9985
7	(0.944759)*hsa-miR-8073+(-1.20451)*hsa-miR-6794-5p+(1.33162)*hsa-miR-3196+(-0.999816)*hsa-miR-6820-5p+(0.540091)*hsa-miR-744-5p+(0.432668)*hsa-miR-4447+(-	0.9894	0.9894	0.9894	0.9986

© 2019 Sudo K et al. *JAMA Network Open.*

	0.0777122)*hsa-miR-3194-3p-12.96439				
7	(0.869211)*hsa-miR-8073+(-1.27205)*hsa-miR-6794-5p+(1.42231)*hsa-miR-3196+(-0.890469)*hsa-miR-6820-5p+(0.469447)*hsa-miR-744-5p+(0.384096)*hsa-miR-4447+(-0.174529)*hsa-miR-1249-5p-12.05034	0.9894	0.9894	0.9894	0.9989
8	(1.02319)*hsa-miR-8073+(-1.08749)*hsa-miR-6794-5p+(1.63503)*hsa-miR-3196+(-1.02075)*hsa-miR-6820-5p+(0.559982)*hsa-miR-744-5p+(0.613777)*hsa-miR-4433a-3p+(-0.277379)*hsa-miR-4734+(0.0347834)*hsa-miR-7641-17.08907	0.9929	0.9929	0.9929	0.9988
8	(1.05478)*hsa-miR-8073+(-1.09539)*hsa-miR-6794-5p+(1.54475)*hsa-miR-3196+(-1.02137)*hsa-miR-6820-5p+(0.610928)*hsa-miR-744-5p+(0.567708)*hsa-	0.9965	0.9894	0.9929	0.9988

© 2019 Sudo K et al. *JAMA Network Open.*

	miR-4433a-3p+(-0.253462)*hsa-miR-4734+(0.0826552)*hsa-miR-128-1-5p-16.89848				
8	(1.02517)*hsa-miR-8073+(-0.881646)*hsa-miR-6794-5p+(1.40786)*hsa-miR-3196+(-0.870833)*hsa-miR-6820-5p+(0.456761)*hsa-miR-744-5p+(-0.174686)*hsa-miR-1247-3p+(-0.216634)*hsa-miR-4688+(0.185067)*hsa-miR-6780b-5p-14.170596	0.9929	0.9894	0.9912	0.9985
8	(1.02218)*hsa-miR-8073+(-0.864953)*hsa-miR-6794-5p+(1.4179)*hsa-miR-3196+(-0.847512)*hsa-miR-6820-5p+(0.557459)*hsa-miR-744-5p+(-0.171894)*hsa-miR-1247-3p+(-0.20082)*hsa-miR-4688+(-0.0246169)*hsa-miR-4667-5p-13.5267	0.9929	0.9894	0.9912	0.9986
8	(0.928114)*hsa-miR-8073+(-1.12068)*hsa-miR-6794-5p+(1.30358)*hsa-miR-3196+(-0.844049)*hsa-miR-6820-	0.9894	0.9965	0.9929	0.9995

© 2019 Sudo K et al. *JAMA Network Open.*

	5p+(0.516218)*hsa-miR-744-5p+(0.436246)*hsa-miR-4447+(-0.0968282)*hsa-miR-3194-3p+(-0.187099)*hsa-miR-7845-5p-12.89114				
8	(0.894265)*hsa-miR-8073+(-1.12991)*hsa-miR-6794-5p+(1.31368)*hsa-miR-3196+(-0.823543)*hsa-miR-6820-5p+(0.506744)*hsa-miR-744-5p+(0.457909)*hsa-miR-4447+(-0.0998605)*hsa-miR-3194-3p+(-0.176987)*hsa-miR-8071-12.88837	0.9894	0.9929	0.9912	0.9991
8	(1.05492)*hsa-miR-8073+(-1.08187)*hsa-miR-6794-5p+(1.72702)*hsa-miR-3196+(-0.976979)*hsa-miR-6820-5p+(0.561999)*hsa-miR-744-5p+(0.602263)*hsa-miR-4433a-3p+(-0.254838)*hsa-miR-4734+(-0.148108)*hsa-miR-4281-17.18846	0.9929	0.9894	0.9912	0.9988
8	(1.04376)*hsa-miR-8073+(-1.0785)*hsa-miR-6794-5p+(1.63137)*hsa-miR-3196+(-0.970992)*hsa-miR-6820-	0.9929	0.9894	0.9912	0.9988

© 2019 Sudo K et al. *JAMA Network Open.*

	5p+(0.548202)*hsa-miR-744-5p+(0.606417)*hsa-miR-4433a-3p+(-0.273145)*hsa-miR-4734+(-0.0269905)*hsa-miR-504-3p-17.17621				
8	(1.04671)*hsa-miR-8073+(-1.08512)*hsa-miR-6794-5p+(1.63315)*hsa-miR-3196+(-0.974758)*hsa-miR-6820-5p+(0.547946)*hsa-miR-744-5p+(0.607058)*hsa-miR-4433a-3p+(-0.266358)*hsa-miR-4734+(-0.0066925)*hsa-miR-3194-3p-17.36915	0.9929	0.9894	0.9912	0.9988
8	(1.05444)*hsa-miR-8073+(-1.06426)*hsa-miR-6794-5p+(1.63579)*hsa-miR-3196+(-0.983745)*hsa-miR-6820-5p+(0.563785)*hsa-miR-744-5p+(0.616715)*hsa-miR-4433a-3p+(-0.274172)*hsa-miR-4734+(-0.0645501)*hsa-miR-6802-5p-17.13668	0.9929	0.9894	0.9912	0.9988
8	(1.04872)*hsa-miR-8073+(-1.06866)*hsa-miR-6794-5p+(1.64535)*hsa-miR-3196+(-0.97453)*hsa-	0.9929	0.9894	0.9912	0.9987

© 2019 Sudo K et al. *JAMA Network Open.*

	miR-6820-5p+(0.547972)*hsa-miR-744-5p+(0.611015)*hsa-miR-4433a-3p+(-0.284385)*hsa-miR-4734+(-0.0385302)*hsa-miR-3131-17.20569				
8	(1.03791)*hsa-miR-8073+(-1.09978)*hsa-miR-6794-5p+(1.63261)*hsa-miR-3196+(-0.986196)*hsa-miR-6820-5p+(0.49976)*hsa-miR-744-5p+(0.605319)*hsa-miR-4433a-3p+(-0.266296)*hsa-miR-4734+(0.0694971)*hsa-miR-4505-17.38343	0.9929	0.9894	0.9912	0.9988
8	(1.0552)*hsa-miR-8073+(-1.12006)*hsa-miR-6794-5p+(1.34768)*hsa-miR-3196+(-0.995383)*hsa-miR-6820-5p+(0.575907)*hsa-miR-744-5p+(0.639412)*hsa-miR-4433a-3p+(-0.307617)*hsa-miR-4734+(0.265763)*hsa-miR-6850-5p-16.40475	0.9965	0.9894	0.9929	0.9988
8	(1.03958)*hsa-miR-8073+(-1.09299)*hsa-miR-6794-5p+(1.6384)*hsa-miR-3196+(-0.96396)*hsa-	0.9929	0.9894	0.9912	0.9988

© 2019 Sudo K et al. *JAMA Network Open.*

	miR-6820-5p+(0.534275)*hsa-miR-744-5p+(0.617925)*hsa-miR-4433a-3p+(-0.269797)*hsa-miR-4734+(-0.0201165)*hsa-miR-6848-5p-17.20927				
8	(1.04137)*hsa-miR-8073+(-1.09255)*hsa-miR-6794-5p+(1.65779)*hsa-miR-3196+(-0.975534)*hsa-miR-6820-5p+(0.538338)*hsa-miR-744-5p+(0.605064)*hsa-miR-4433a-3p+(-0.263259)*hsa-miR-4734+(-0.0354932)*hsa-miR-762-17.08333	0.9929	0.9894	0.9912	0.9988
8	(1.06298)*hsa-miR-8073+(-1.04732)*hsa-miR-6794-5p+(1.7333)*hsa-miR-3196+(-0.893881)*hsa-miR-6820-5p+(0.580881)*hsa-miR-744-5p+(0.59551)*hsa-miR-4433a-3p+(-0.238889)*hsa-miR-4734+(-0.322119)*hsa-miR-4656-17.8277	0.9929	0.9894	0.9912	0.9989

© 2019 Sudo K et al. *JAMA Network Open*.

8	(1.0969)*hsa-miR-8073+(-0.975023)*hsa-miR-6794-5p+(1.6108)*hsa-miR-3196+(-0.89195)*hsa-miR-6820-5p+(0.569533)*hsa-miR-744-5p+(0.592897)*hsa-miR-4433a-3p+(-0.291404)*hsa-miR-4734+(-0.19194)*hsa-miR-1247-3p-17.38892	0.9965	0.9859	0.9912	0.9992
8	(1.03146)*hsa-miR-8073+(-1.09937)*hsa-miR-6794-5p+(1.59149)*hsa-miR-3196+(-0.947698)*hsa-miR-6820-5p+(0.547429)*hsa-miR-744-5p+(0.633284)*hsa-miR-4433a-3p+(-0.303073)*hsa-miR-4734+(-0.138804)*hsa-miR-6870-5p-15.58493	0.9965	0.9859	0.9912	0.9988
8	(1.03835)*hsa-miR-8073+(-1.08336)*hsa-miR-6794-5p+(1.63855)*hsa-miR-3196+(-0.960421)*hsa-miR-6820-5p+(0.542553)*hsa-miR-744-5p+(0.612356)*hsa-miR-4433a-3p+(-0.274082)*hsa-miR-4734+(-0.0256252)*hsa-miR-3928-3p-17.2588	0.9929	0.9894	0.9912	0.9988

© 2019 Sudo K et al. *JAMA Network Open.*

8	(1.06456)*hsa-miR-8073+(-1.04588)*hsa-miR-6794-5p+(1.58298)*hsa-miR-3196+(-0.929148)*hsa-miR-6820-5p+(0.583733)*hsa-miR-744-5p+(0.594611)*hsa-miR-4433a-3p+(-0.277877)*hsa-miR-4734+(-0.129398)*hsa-miR-365a-5p-16.70549	0.9929	0.9859	0.9894	0.9987

© 2019 Sudo K et al. *JAMA Network Open.*

eTable 2. Independent Association Between the Six-miRNA Panel (EC Index) and the Presence of Esophageal Squamous Cell Carcinoma

	Univariable analysis			Multivariable analysis	
	odds ratio	(95% CI)		odds ratio	(95% CI)
EC index (per 1 increase)	36.2	22.9–57.1		38.2	23.1–63.3
Age (per 10 years)	1.06	0.98–1.14		1.52	1.19–1.93
Gender (men)	6.05	4.41–8.29		8.87	3.98–19.7

CI: confidence interval

© 2019 Sudo K et al. *JAMA Network Open.*

eFigure 1. Dot Plots for Six miRNAs in ESCC and Non-cancer Controls in the Training Set

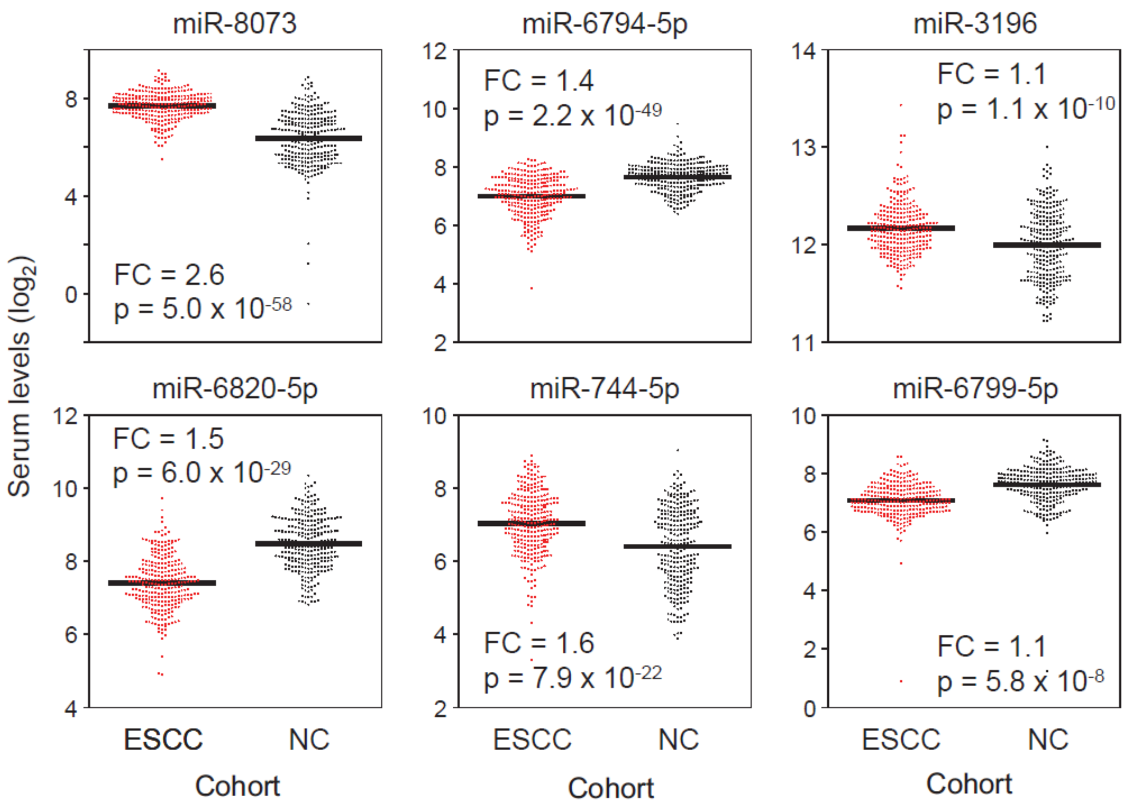

ESCC and non-cancer control samples in the training set were plotted. ESCC: esophageal squamous cell carcinoma; NC: non-cancer; FC: fold change; p value: two-sided Student t-test.

© 2019 Sudo K et al. *JAMA Network Open.*

eFigure 2. ROC Analysis of Individual miRNAs for Distinguishing ESCC From Non-cancer Controls in the Training Set

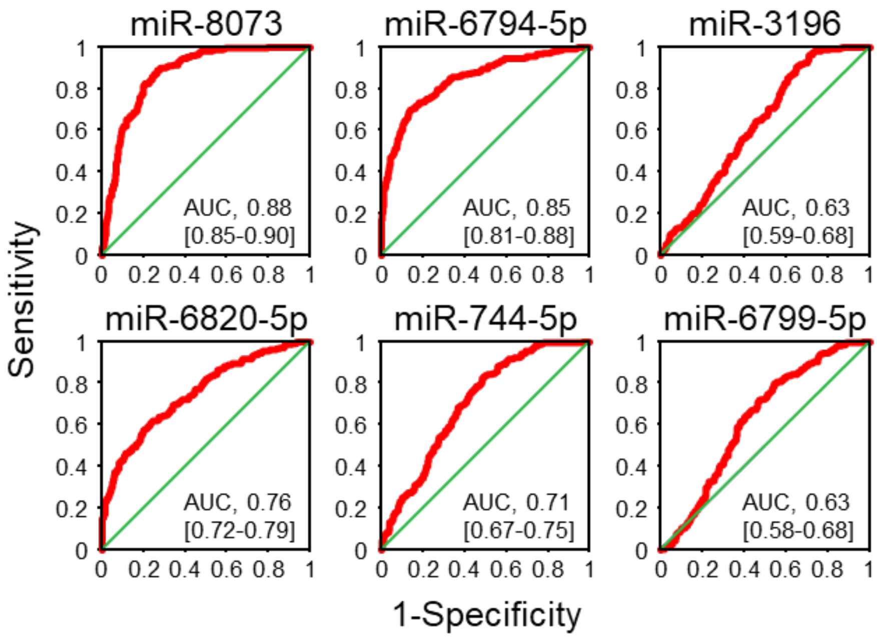

The AUC and 95% CI are shown in the plot. AUC: area under the receiver operating curve.

© 2019 Sudo K et al. *JAMA Network Open*.
